# An integrative review of the side effects related to the use of magnesium sulfate for pre-eclampsia and eclampsia management

**DOI:** 10.1186/1471-2393-13-34

**Published:** 2013-02-05

**Authors:** Jeffrey Michael Smith, Richard F Lowe, Judith Fullerton, Sheena M Currie, Laura Harris, Erica Felker-Kantor

**Affiliations:** 1 Jhpiego, 1776 Massachusetts Ave. NW #300, Washington, DC, 20036, USA; 2 Venture Strategies Innovations, 19200 Von Karman Avenue, Suite 400, Irvine, CA, 92612, USA; 3 Independent Consultant; Professor (Ret.) University of California San Diego, 7717 Canyon Point Lane, San Diego, CA, 92126, USA; 4 School of Public Health, University of California, Berkeley, 50 University Hall, #7360, Berkeley, CA, 94720, USA; 5 Johns Hopkins Bloomberg School of Public Health, 615 N. Wolfe Street, Baltimore, MD, 21205, USA

**Keywords:** Pre-eclampsia, Eclampsia, Magnesium sulfate, Side effect

## Abstract

**Background:**

Pre-eclampsia/eclampsia is one of the most common causes of maternal and perinatal morbidity and mortality in low and middle income countries. Magnesium sulfate is the drug of choice for prevention of seizures as part of comprehensive management of the disease. Despite the compelling evidence for the effectiveness of magnesium sulfate, concern has been expressed about its safety and potential for toxicity, particularly among providers in low- and middle-income countries. The purpose of this review was to determine whether the literature published in these global settings supports the concerns about the safety of use of magnesium sulfate.

**Methods:**

An integrative review of the literature was conducted to document the known incidences of severe adverse reactions to magnesium sulphate, and specific outcomes of interest related to its use. All types of prospective clinical studies were included if magnesium sulfate was used to manage pre-eclampsia or eclampsia, the study was conducted in a low- or middle-income country, and the study included the recording of the incidence of any adverse side effect resulting from magnesium sulfate use.

**Results:**

A total of 24 studies that compared a magnesium sulfate regimen against other drug regimens and examined side effects among 34 subject groups were included. The overall rate of absent patellar reflex among all 9556 aggregated women was 1.6%, with a range of 0-57%. The overall rate of respiratory depression in 25 subject groups in which this outcome was reported was 1.3%, with a range of 0–8.2%. Delay in repeat administration of magnesium sulfate occurred in 3.6% of cases, with a range of 0-65%. Calcium gluconate was administered at an overall rate of less than 0.2%. There was only one maternal death that was attributed by the study authors to the use of magnesium sulfate among the 9556 women in the 24 studies.

**Conclusion:**

Concerns about safety and toxicity from the use of magnesium sulfate should be mitigated by findings from this integrative review, which indicates a low incidence of the most severe side effects, documented in studies that used a wide variety of standard and modified drug regimens. Adverse effects of concern to providers occur infrequently, and when they occurred, a delay of repeat administration was generally sufficient to mitigate the effect. Early screening and diagnosis of the disease, appropriate treatment with proven drugs, and reasonable vigilance for women under treatment should be adopted as global policy and practice.

## Background

Pre-eclampsia/eclampsia (PE/E) is a life-threatening multisystem disorder affecting 2 - 8% of all pregnancies worldwide [1,2] that has substantial effect on maternal and newborn health. PE/E is one of the most common causes of maternal and perinatal morbidity and mortality in low and middle income countries [3]. Globally, approximately 63,000 women die each year of PE/E which accounts for an estimated 9% of maternal deaths in Asia and Africa and about one-quarter of maternal deaths in Latin America and the Caribbean [1,3-6].

Manifestations of severe pre-eclampsia should be treated in accord with World Health Organization recommendations [7]. Comprehensive management of the disease includes vigilant monitoring of the woman and fetus, management of acute hypertension and prevention of seizures in women with pre-eclampsia, and prevention of recurrent seizures in women with eclampsia. The definitive treatment of PE/E is delivery of the fetus.

Magnesium sulfate is the drug of choice for prevention of seizures in the pre-eclamptic woman, or prevention of recurrence of seizures in the eclamptic woman, as demonstrated in two large clinical studies. In 1995, the Eclampsia Trial Collaborative Group reported that when magnesium sulfate was used for treatment the risk of recurrent convulsions in women with eclampsia was reduced by 52% when compared with diazepam, and by 67% when compared with phenytoin [8]. In 2002, the Magpie trial reported that women with severe pre-eclampsia given magnesium sulfate had a 58% lower risk of developing eclampsia compared to the placebo group [9]. Findings from a recent Cochrane review [10] also support the use of magnesium sulfate as the drug of choice. Although the precise mechanism of action is unclear, magnesium sulfate appears to have a peripheral site of action at the neuromuscular junction and does not cross the intact blood brain barrier [11]. Pritchard showed that magnesium serum concentration required for eclampsia prevention or treatment should be higher than normal serum levels, and suggested that therapeutic concentration should be between 4 and 7 mEq/L [12].

Magnesium sulfate is associated with several minor side effects such as a feeling of warmth, flushing, nausea and vomiting, muscle weakness, somnolence, dizziness, and irritation at the injection site. More serious side effects are rare but include the loss of the patellar reflex (typically occurring at a serum concentration of 8 -10 mEq/L) and respiratory depression (>13 mEq/L) [11,13].

Routine monitoring of a woman undergoing magnesium sulfate therapy includes simple assessment of neurologic status (level of alertness and patellar reflexes), respiratory rate and urinary output [14]. Typical management of the more serious side effects includes heightened monitoring, delay in administration of next dose or suspension of magnesium sulfate therapy. Oliguria is an element of the disease process [reduced clearance by the kidneys], and not an adverse effect of magnesium sulfate use. Because magnesium is cleared by the kidneys, oliguria of less than 30 cc per hour is used as a determinant for withholding a scheduled dose, in order to prevent toxic levels. If serious toxicity is suspected, and immediate counteraction of magnesium is desired, calcium gluconate can be administered to counteract the effect of magnesium levels that are well above the therapeutic range [13].

Despite the compelling evidence for the effectiveness of magnesium sulfate concern has been expressed about the safety of its administration and use, particularly in clinical environments where the capacity for patient monitoring is limited. These concerns can constrain initiation of treatment for all women with indicated need, or may impede sustaining therapy over the recommended timeline established for the particular regimen, once treatment has been initiated [15,16].

The purpose of this review was to determine whether the published literature from low- and middle-income countries supports the concerns about the safety of use of magnesium sulfate. The review identifies the frequency of severe adverse reactions when the drug is used according to a recommended protocol, and the frequency of the need for intervention as a result of those reactions.

## Methods

### Research questions

We conducted an integrative review of the literature to answer the following research questions about clinical outcomes, when magnesium sulfate is correctly administered according to one of the three commonly used regimens, or a modification of one of the three regimens. For women who undergo therapy with magnesium sulfate for severe pre-eclampsia or eclampsia:

· What are the known incidences of side effects of absent patellar reflex and respiratory depression?

· With what frequency do providers report use of calcium gluconate to counteract the effects of magnesium sulfate in response to detected side effects?

· How often are doses of magnesium sulfate skipped or delayed in response to the development of side effects?

· How many maternal deaths of women with severe pre-eclampsia and eclampsia have been reported to be attributed to toxicity of magnesium sulfate rather than from manifestations of the underlying disease?

The use of an integrative review expands the variety of research designs that can be incorporated within a review’s inclusion criteria [17,18], and allows the incorporation of both qualitative and quantitative information. This review did not attempt to reanalyze or combine primary data, nor did it make determinations regarding the quality of original studies.

### Literature search

MEDLINE [via PubMed], Embase, and The Cochrane Library were searched for relevant studies. Reference lists in relevant journal articles were also searched. Searches were limited to the years 1980 through February 2012. No language restrictions were enforced. For PubMed, medical subject headings [MeSH] used were *magnesium sulfate*, *pre-eclampsia and eclampsia.* The terms *sulfamag, sulmetin, sulmetine, MgSO*_*4*_*, magnesium sulphate, pre-eclamptic, preeclamptic, eclamptic, EPH Complex, EPH Gestosis, EPH, Hypertension Edema Proteinuria Gestosis, Pregnancy Toxemia, puerperal tetany* were also used as search terms in combinations with the MeSH search terms. The same search terms were used for Embase.

Articles were first screened by review of the title. Selected articles were further screened by review of the abstract. The final chosen articles were read and the desired data summarized. The criteria for inclusion were:

· magnesium sulfate was used to manage pre-eclampsia or eclampsia in a prospective clinical study;

· the study was conducted in a low- or middle-income country [19];

· the study included the recording of the incidence of any adverse side effect resulting from magnesium sulfate use (sluggish or absent patellar reflexes or respiratory depression), and may have included report of oliguria or use of calcium gluconate.

Studies were excluded if they were medical record reviews, had been conducted in high-income countries (where treatment and monitoring approaches may differ) or did not specifically record the incidence of side effects related to magnesium sulfate use. All types of clinical studies were included.

A specific data extraction form was designed for the study. The form reflected terminology and definitions that appeared in the various reports (e.g., imminent eclampsia), and data were later recoded to meet commonly accepted clinical definitions (e.g., imminent eclampsia was re-categorized as pre-eclampsia, which included both mild and severe manifestations). Data were extracted only for women who received magnesium sulfate as a therapeutic intervention in prospective observational studies, or as a treatment arm in comparative trials.

For eligible studies, one reviewer (LH) carried out data extraction. RL, EFK, and JF independently repeated data extraction on randomly-assigned samples of studies, achieving a 100% second review. Discrepancies were resolved through consultation with a fourth reviewer (JMS). Data were entered into Microsoft Excel [Microsoft Corporation] for analysis.

Information about each of the side effects of our specific interest was not present in every published study, and data on the incidence of an outcome was recorded only when specifically mentioned in an article. We made the assumption in our extraction protocol that if a side effect was not reported, it did not occur. Therefore, the denominator for the incidence of given adverse effects was the sum of all subjects in the reviewed studies.

## Results

**Figure 1:**
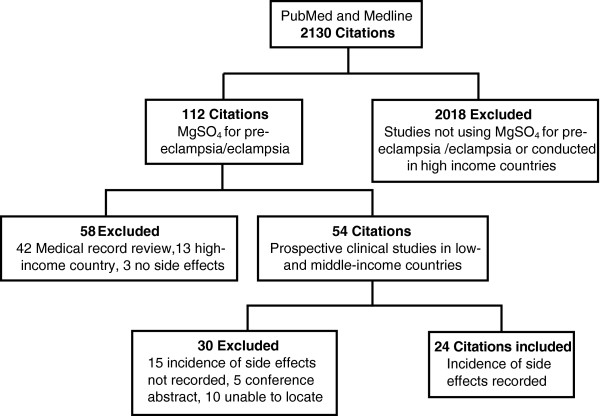
Selection of studies included in the review.

Of the 54 clinical studies conducted in low- and middle-income countries identified, 24 were included in the final analysis (Figure 1). Of the 30 studies that were excluded, 15 did not record the incidence of side effects, five were available as conference abstracts only, and ten studies were unable to be located despite searching several libraries and contacting authors where possible.

The final studies that were reviewed included randomized and non-randomized trials, either open or blinded (Table 1). Most studies were randomized controlled trials in which a magnesium sulfate regimen was compared against other drug regimens, or another magnesium sulfate regimen. Prospective cohort trials typically involved just one magnesium sulfate regimen. A single case control study was identified.

**Table 1:** Characteristics of the studies and regimens used

**Author/year**	**Country**	**Regimen**	**Modification from standard regimen**	**Study Design**	**Randomized (Y/N)**
**PRITCHARD REGIMENS **[12]					
Alauddin 2011 [20]	India	P	N/A	Prospective cohort	N
Bhattarcharjee 2011 [21]	India	P	N/A	RCT	Y
		Pm	4 g loading; 6 g/8 h maintenance		
Bhattacharya 2010 [22]	India	P	N/A	Case Control	N
Bhalla 1994 [23]	India	Pm	12 g loading	RCT	Y
Chowdhury 2009 [24]	India	P	N/A	RCT	Y
		Zm	0.6 g/h maintenance		
Chissell 1994 [25]	South Africa	P	N/A	RCT	Y
		Zm	6 g loading 2 g/h maintenance		
Crowther 1990 [26]	Zimbabwe	P	N/A	RCT	Y
Ekele 2005 [27]	Nigeria	P	N/A	Prospective cohort	N
Malapaka 2011 [28]	India	P	N/A	RCT	Y
		Pm	4 g loading; 2 g/3 h maintenance		
Mahajan 2009 [29]	India	Pm (A)	10 g loading	Prospective cohort	N
		Pm (B)	6 g loading		
Manorot 1996 [30]	Thailand	P	N/A	RCT	Y
		Z	N/A		
Mundle 2011 [31]	India	P	N/A	RCT	Y
		Z	N/A		
Shoaib 2009 [32]	Pakistan	P	N/A	Prospective cohort	N
		Pm	No maintenance dose		
**ZUSPAN REGIMENS **[33]					
Aali 2007 [34]	Iran	Zm	2 g/h maintenance	Prospective cohort	N
Belfort 2003 [35]	14 countries	Z or Zm	6 g loading; 2 g/h maintenance	RCT	Y
Coetzee 1998 [36]	South Africa	Z	N/A	RCT	Y*
Dayicioglu 2003 [37]	Turkey	Zm	4.5 g loading; 1.8 g/h maintenance	Prospective cohort	N
Deshmukh 1985 [38]	Bangladesh	Z or Zm	1 g/h maintenance for 8 h	Prospective cohort	Y
Dommisse 1988 [39]	South Africa	Z or Zm	2 g/h maintenance	RCT	Y
Phuapradit 1993 [40]	Thailand	Zm	5 g loading	Prospective cohort	N
**PRITCHARD OR ZUSPAN**					
Altman 2002 [9]	33 countries	P or Z	N/A	RCT	Y*
Eclampsia Trial Collaborative Group 1995 [8]	9 countries	P or Z^1^	N/A	RCT	Y
		P or Z^2^	N/A	RCT	Y
**DHAKA REGIMENS **[41]					
Begum 2001 [41]	Bangladesh	D	N/A	Prospective cohort	N
Shilva 2007 [42]	India	D	N/A	RCT	Y*
		Dm	12 g loading		

P = Pritchard; Z = Zuspan; D = Dhaka. Use of “m” after the regimen indicates a modified regimen.

^1^ compared against diazepam; ^2^ compared against phenytoin.

* blinded.

In the 24 studies, we identified 34 distinct subject groups that were administered magnesium sulfate, comprising a total of 9556 women. The women in these groups received one of three common magnesium sulfate regimens (Table 2) or a modification of one of these standard regimens. In total 16 different magnesium sulfate regimens were used in the 24 studies. Eleven subject groups received the standard Pritchard regimen [12] and six groups received one of six different modified Pritchard regimens. Three groups received the standard Zuspan regimen [33] and five groups received one of six Zuspan modified regimens. In three groups, some women received either the Zuspan or one of the six modified Zuspan regimens. Two groups received the Dhaka regimen [41] and one a modified Dhaka regimen, and in three subject groups women received either the Pritchard or Zuspan regimen.

**Table 2:** Regimens of magnesium sulfate most commonly used in studies

	**Pritchard**	**Zuspan**	**Dhaka**
**Loading**	4 g in 20 mL (20% solution) administered IV over 15-20 minutes, followed by 5 g in 10 mL solution (50%) IM injection in each buttock.	4 g in 20 mL (20% solution) administered IV over 15-20 minutes.	4 g in 20 mL (20% solution) administered IV over 15-20 minutes followed by 3 g in 6 mL (50% solution) IM injection in each buttock.
**Maintenance**	5 g in 10 mL (50% solution) IM injection every 4 hours in alternate buttocks.	1 g/hour IV infusion	2.5 g in 5 mL (50% solution) IM injection every 4 hours in alternate buttocks.
**Duration**	24 hours after last convulsion or delivery, whichever occurs later		
**Modifications to standard regimens**	Modifications included a reduction in the loading dose, or an increase or decrease in the maintenance dose. Some studies also reduced the length of time for which therapy was administered and some stopped therapy after the loading dose.		
	Two factors affect the amount of magnesium sulfate administered in a course of treatment:		
	a) duration of labor between the administration of the loading dose and delivery; b) additional convulsions following the loading dose.		

Tables 3, 4 and 5 show the numbers of women enrolled in magnesium sulfate treatment groups, and the seven outcomes of interest. These included affected (either sluggish or absent) patellar tendon reflex, respiratory depression of less than 16 respirations per minute, oliguria of less than 30cc per hour, a decision to skip or delay the administration of the next scheduled dose, administration of calcium gluconate for perceived toxicity, all maternal deaths and maternal deaths considered to be due to magnesium sulfate toxicity. The calculated overall incidence rates for the outcomes of interest in all 9556 women are presented in Table 6.

**Table 3:** Enrollment and outcomes in studies using Pritchard or modified Pritchard regimens

	**Enrollment N**			**Outcomes N (%)**						
**Study**	**Subjects**	**Pre-eclampsia**	**Eclampsia**	**Affected patella reflex**	**Respiratory depression**	**Oliguria**	**Skipped dose**	**Calcium gluconate**	**Maternal death**	
									**All**	**Due to MgSO**_**4**_
**PRITCHARD**										
Alauddin	459	0	459	2	0	4	6	0	17	0
Bhattacharya	50	50	0		2	8	10		0	0
Bhattacharjee	70	0	70			0			3	0
Chowdhury	480	0	480	15	0	15	15		24	0
Chissell	9	9		0	0	0	0		0	0
Crowther	24	0	24		0	3			1	0
Ekele	19	0	19	0	0	1	1	0	0	0
Malapaka	54	16	38	19	1	19	33^1^		1	1
Manorot	25	25	0	0	0	0	0	0	0	0
Mundle	153	153	0				12^2^		0	0
Shoaib	50	50			0				0	0
**SUBTOTALS**	**1393**	**303**	**1090**	**36 (2.6)**	**3 (0.2)**	**50 (3.6)**	**77 (5.5)**	**0 (0)**	**46 (3.3)**	**1 (0.1)**
**PRITCHARD MODIFIED**										
Bhattacharjee	67		67	0	0	0	0		1	0
Bhalla	45	0	45		0	2		0	0	0
Malapaka	72	37	35	5	0	7	12^5^		1	0
Mahajan A ^3^	58	0	58	18	0	0	18	0	0	
Mahajan B ^4^	37	0	37	21	0	3	24	0	0	
Shoaib	50	50			0				0	0
**SUBTOTALS**	**329**	**87**	**242**	**44 (13.4)**	**0 (0)**	**12 (3.7)**	**54 (16.4)**	**0 (0)**	**2 (0.6)**	**0 (0)**
**TOTALS**	**1722**	**390**	**1332**	**80 (4.7)**	**3 (0.2)**	**62 (3.6)**	**131 (7.6)**	**0 (0)**	**48 (2.8)**	**1 (0.1)**

^1^ 14 stopped due to signs of magnesium sulfate toxicity; ^2^ 10 stopped treatment due to woman’s request, side effects, oliguria or renal failure, or signs of toxicity and 2 due to provider error or provider preference (Personal communication); ^3^Group A received 10 g loading dose; ^4^Group B received 6 g loading dose; ^5^ 1 stopped due to signs of magnesium sulfate toxicity.

**Table 4:** Enrollment and outcomes in studies using Zuspan or modified Zuspan regimens

	**Enrollment N**			**Outcomes N (%)**						
**Study**	**Subjects**	**Pre-eclampsia**	**Eclampsia**	**Affected patella reflex**	**Respiratorydepression**	**Oliguria**	**Skipped dose**	**Calcium gluconate**	**Maternal death**	
									**All**	**Due to MgSO**_**4**_
**ZUSPAN**										
Belfort ^1^	831	831	0			55			0	0
Coetzee	345	345	0		1			1 *	0	0
Deshmukh ^2^	18	12	6	0	1	0	0	1	0	0
Dommisse ^3^	11	0	11	0	0	0			0	0
Manorot	25	25	0	0	0	0	0	0	0	0
Mundle	147	147	0				13^4^		0	0
**SUBTOTALS**	**1377**	**1360**	**17**	**0 (0.0)**	**2 (0.15)**	**55 (4.0)**	**13 (0.9)**	**2 (0.15)**	**0 (0)**	**0 (0)**
**ZUSPAN MODIFIED**										
Aali	50	46	4	1			0	0	0	0
Chowdhury	150	0	150	0	0	0			5	0
Chissell	8	8	0	1	0	1	1		0	0
Dayicioglu	194	194	0					1	0	0
Phuapradit	44	44	0	0			0			
**SUBTOTALS**	**446**	**292**	**154**	**2 (0.5)**	**0 (0)**	**1 (0.2)**	**1 (0.2)**	**1 (0.2)**	**5 (1.1)**	**0 (0)**
**TOTALS**	**1823**	**1652**	**171**	**2 (0.01)**	**2 (0.1)**	**56 (3.1)**	**14 (0.8)**	**3 (0.2)**	**5 (0.3)**	**0 (0)**

^1^ Subjects received either Zuspan or Zuspan modified; ^2^ Eclamptics received Zuspan regimen; pre-eclamptics received modified Zuspan regimen; ^3^ Five eclamptics received Zuspan regimen and six received a modified Zuspan regimen; ^4^ 6 stopped treatment due to woman’s request, side effects, oliguria or renal failure, or signs of toxicity and 7 due to provider error or provider preference (Personal communication); * Calcium gluconate administered after a magnesium sulfate dosing error caused respiratory depression.

**Table 5:** Enrollment and outcomes in studies using Pritchard or Zuspan, or any Dhaka regimen

	** Enrollment N**			**Outcomes N (%)**						
**Study**	**Subjects**	**Pre-eclampsia**	**Eclampsia**	**Affected patella reflex**	**Respiratory depression**	**Oliguria**	**Skipped dose**	**Calcium gluconate**	**Maternal death**	
									**All**	**Due to MgSO**_**4**_
**PRITCHARD OR ZUSPAN**										
Altman ^1^	5055	5055	0	59 ^4^	51^4^	114	187^5^	14	11	0
Eclampsia Collab. Trial A ^2^	453	0	453		35				17	
Eclampsia Collab. Trial B ^3^	388	0	388		32				10	
**TOTAL**	**5896**	**5055**	**841**	**59 (1.0)**	**118 (2.0)**	**114 (1.9)**	**187 (3.2)**	**14 (0.2)**	**38 (0.6)**	**0 (0)**
**DHAKA (ALL)**										
Begum	65	0	65	5	0	0			0	0
Shilva	25	0	25	8		5	13^7^		0	0
Shilva ^6^	25	0	25	2		1	3^8^		0	0
**TOTAL**	**115**	**0**	**115**	**15 (13)**	**0 (0)**	**6 (5.2)**	**16 (13.9)**	**0 (0)**	**0 (0)**	**0 (0)**

^1^ Subjects received either Pritchard or Zuspan regimen; ^2^ Study of Pritchard or Zuspan regimen vs diazepam; ^3^ Study of Pritchard or Zuspan regimen vs phenytoin; ^4^ 4 Women had respiratory depression and absent tendon reflex; ^5^ Includes 114 women who stopped treatment because of oliguria or renal failure, 26 women who stopped treatment because of respiratory depression or arrest, and 47 women who stopped treatment because of absent tendon reflex; ^6^ Received Dhaka modified regimen; ^7^ 8 Skipped because loss of knee-jerk and 5 because of oliguria; ^8^ 2 Skipped because loss of knee-jerk and 1 because of oliguria.

**Table 6:** Overall outcome rates for all studies in 9556 subjects

	**Affected patella reflex**	**Respiratory depression**	**Oliguria**	**Skipped dose**	**Calcium gluconate use**	**Maternal death**	
						**All**	**Due to MgSO**_**4**_
Number of events	156	123	238	348	17	91	1
Incidence (%)	1.6	1.3	2.5	3.6	0.18	0.9	0.01
Range of incidence%^1^	0 – 57	0 - 8.2	0 – 35	0 – 65	0 - 0.29	0 – 5	0-0.01

^1^ Based on incidence of the event reported in each study.

### Incidence of affected patellar reflex

Absent patellar reflex was specifically reported for 20 subject groups. The overall incidence among all 9556 aggregated women was 1.6%, with the incidence rate ranging from 0-57%. Three studies containing less than 60 women in each subject group recorded incidences ranging from 35%-57% [28,29,42], while the incidence was only 1.2% for the 5055 subjects in the Magpie trial [9].

### Incidence of respiratory depression

Respiratory depression was specifically reported for 25 subject groups. The overall incidence among all 9556 women was 1.3%, with the incidence ranging from 0–8.2%. The highest incidences (7.7% and 8.2%) were reported for the two magnesium sulfate groups in the Eclampsia Collaborative Trial which included a total of 841 subjects [8]. In the three studies that reported very high incidences of absent patellar reflex, the incidence of respiratory depression was less than 1% [28,29,42].

### Frequency with which doses of magnesium sulfate were skipped or delayed

The skipping or delaying of a dose of magnesium sulfate was usually done in the presence of an adverse side effect (noted above), but in some studies, was also done following provider or patient preference. This outcome was reported for 22 subject groups. Repeat administration of magnesium sulfate was skipped or delayed in 348 women out of the total of 9556 in all trials, for a rate of 3.6%, with the rate ranging from 0-65%. The highest rates ranging from 20-65% were reported in studies in which subject groups were smaller than 70 [21,28,29,42], while the largest study in 5055 women reported a rate of 3.7% [9].

### Frequency of use of calcium gluconate

The use of calcium gluconate was only reported for 12 of the 34 subject groups. The drug was administered only 17 times, resulting in an overall rate among 9556 women of less than 0.2%. In one study, calcium gluconate was administered to a woman following a dosing error which resulted in administration of 4 g of magnesium sulfate in one hour instead of four hours. The majority of the events (n = 14) occurred in the Magpie trial [9].

### Maternal deaths attributable to magnesium sulfate

There was only one maternal death that was attributed by the study authors to the use of magnesium sulfate in the 24 studies [28]. The authors reported that the death was caused by severe respiratory depression and the woman’s serum magnesium level was reported at 24 mEq/L, well above the therapeutic limits for prevention of eclamptic seizures.

## Discussion

Magnesium sulfate therapy for treatment of eclampsia is cited as one of the 56 essential evidence-based interventions that together could potentially eliminate the untimely deaths of 358,000 women and 7.6 million children in low- and middle-income countries [43]. The drug currently appears on 50% of the essential medicines lists from 89 countries [44] and has recently been included as one of 13 essential commodities in the UN Commission on Essential Drugs for Maternal and Child Health [45].

Despite global consensus about its effectiveness and safety, clinicians in many countries continue to demonstrate reluctance to use the drug in their management of women with severe pre-eclampsia or eclampsia. Concerns have been raised about both anticipated side effects of the drug, such as warmth, somnolence and neurological depression, as well as adverse side effects relating to potential toxicity of the drug, including absent patellar reflexes and respiratory depression that can lead to cardiac depression and death.

The pharmacological effect of the drug is minor to moderate neurological depression, typically manifested as diminished reflexes and somnolence. Some clinicians equate this with impending toxicity despite information which demonstrates that serum levels where neurological depression is seen (4 – 7 mEq/L) are substantially lower than serum levels that are associated with the toxicity-related side effects of absent patellar reflexes (>10 mEq/L), respiratory depression (>13 mEq/L) or cardiac (25 mEq/L) dysfunction [46]. The concerns about adverse side effects have sometimes resulted in a reluctance to use magnesium sulfate without the availability of the antidote calcium gluconate, or ability to measure serum magnesium levels. This reluctance has also contributed to the persistence of the use of diazepam or lytic cocktails, which are known to be both inferior in effectiveness and without clinical antidote. This integrative review provides a summary of the incidence of adverse side effects and seeks to clarify the safety profile of magnesium sulfate when used for prevention or treatment of eclamptic seizures.

McDonald et al. [47] recently conducted a systematic review of maternal and infant outcomes following magnesium sulfate therapy, and addressed the issue of drug safety. Findings from that systematic review, which included studies from both developed and developing countries indicated that the use of magnesium sulfate for pre-eclampsia reduced the risk for progression of the disease, and that use of the drug among patients with eclampsia was associated with lower risks of maternal death, recurrent seizures and major morbidity. Other clinical reviews and toxicology studies also indicate that the drug is safe to use, for indicated purposes, in recommended dosages and according to standardized protocol for administration and monitoring of the drug [13,20,48,49]. Findings from this integrative review enhance what is known about the incidence and severity of side effects when the drug is used appropriately.

We made the assumption in our extraction protocol that if a side effect or event was not reported it did not occur. Five outcomes (Tables 3, 4 and 5) were specifically mentioned in the methods section of the various articles at a range of 38% (use of calcium gluconate) to 75% (respiratory depression). Additionally, several studies reported on one or more of these same outcomes, even if not specifically noted in the study methods. In the interest of reviewing the potential for bias in our methodological approach we computed the incidence of side effects/outcomes, using only the subset of studies in which each of these was specifically reported. Accordingly, the calculated incidence of each side effect/outcome in this subset was higher. We acknowledge that our assumption that the event itself did not occur (rather than was simply not reported) may have resulted in an underestimate of the actual incidence of any specific side effect, a limitation of this study.

Across all studies in which the occurrence of a side effect was reported, the incidence of affected patellar reflex (sluggish or absent) did not exceed 1.6% and the incidence of respiratory depression was 1.3%. Although in four study groups totaling less than 150 subjects there were reported incidences of affected patellar reflex of up to 57%, only one subject was reported as experiencing respiratory depression, a more serious symptom that is observed with higher serum concentrations of magnesium [28,29,42]. These same three studies also reported the highest frequency of skipped doses, from 30-60%, but some doses were skipped due to provider or patient preference.

In the Magpie study which had the largest study group of over 5000 subjects, the incidence of the two adverse side effects was roughly the same, around 1% [9]. This trial also reported that 187 subjects skipped doses but in over 60% of those subjects (n =114), the dose was skipped due to oliguria or renal failure, a manifestation of the disease and not due to magnesium sulfate. In the study by Mundle [31], a total of 25 women in the two groups had scheduled doses of magnesium withheld; 10 due to signs of toxicity (usually depressed tendon reflexes), one due to oliguria and 14 due to provider or patient preference or other side effects [Personal communication Bracken, July 11, 2012].

The relatively more common perception of side effects and higher frequency of skipped doses among studies with smaller populations are in contrast to the lower frequency of skipped doses among the studies with larger populations. This may suggest that with greater use of the drug and the experience that comes with managing a larger number of women on magnesium clinicians become more skilled at evaluating the patient and understanding the normal response to the drug.

Calcium gluconate use was extremely infrequent. It would require 555 women to be treated in order to experience one situation in which administration of calcium gluconate would be needed (Table 7). Given that the studies reviewed were prospective clinical trials of magnesium sulfate, the reviewers assumed that even if availability of calcium gluconate was not explicitly stated by the authors, it was present as part of prudent research implementation. Therefore we also assume that the low use reflects an infrequent need for its use as an antidote to the magnesium rather than due to its unavailability [50,51].

**Table 7:** Estimates of clinical impact

	**Affected patellar reflex**	**Respiratory depression**	**Skipped or delayed dose**	**Calcium gluconate use**
Incidence amongst 9556 women	1.6%	1.3%	3.6%	0.18%
Number of pre-eclamptic/eclamptic women needed to treat to experience one incidence (i.e., number needed to harm)	61	77	27	555
Scenario: A hospital delivers 5000 women annually. Assuming a rate of PE/E of 5%, 250 women annually will require treatment with magnesium sulfate.				
Frequency of 1 case (months)	2.9 months	3.7 months	1.3 months	26.7 months

Almost half of the studies were conducted in India and Bangladesh and many of these compared standard regimens with lower dose regimens. In these countries, it is felt that lower doses are justified based on the smaller size (lower weight) of the women. Studies do show that lower doses may be equally as effective as the standard regimens in preventing seizures for women with severe pre-eclampsia [21,24,28,29], with resultant fewer side effects. Two studies indicated that the lower dose regimen was less effective when used for treatment of women who had eclampsia [28,42].

Still, if concerns about drug toxicity can be set aside, additional concerns continue to be expressed about the impact on health systems and services, when magnesium sulfate is prescribed as therapy. Particular concerns are expressed more frequently by health care administrators and providers who serve in lower-resource settings [52-54]. It is for this reason that our report excluded studies conducted in high-income countries. Treatment approaches may differ in higher-resource settings (e.g., use of intravenous infusion pumps, rather than intramuscular administration); and lower patient/staff ratios typically enable more vigilant patient monitoring in these settings.

The chronic shortage of health care personnel to provide the one-to-one observation and support of women is frequently cited as a barrier to provision of quality care. Task shifting (task sharing) among various professional and lesser-skilled cadres may contribute to the solution of the health workforce problem [15,16,50,55-57]. Still, in a hospital that conducts 5,000 deliveries annually, and assuming a rate of PE/E of 5%, under normal use of magnesium sulfate regimens for women with PE/E, and without medication errors, a case of affected patellar reflexes would be experienced once every three months and a case of respiratory depression just less than once every four months. Use of calcium gluconate would be required once every 27 months, making it a rare event (Table 7).

Based on incidence of the two adverse effects, 61 – 77 pre-eclamptic or eclamptic women would need to be observed during treatment in order to identify one adverse effect of magnesium sulfate, leading to the decision to delay or skip a dose (Table 7). However, the results show that a dose would be skipped every 27 women observed, supporting the finding that some doses were skipped for reasons other than experiencing side effects of magnesium sulfate administration.

Measuring and recording urine output should be straightforward in most countries as it is recommended that all women with PE/E should be catheterized [14], and therefore monitoring should not create high demand on the time of health care personnel. There is also little support for the need to conduct laboratory analyses of urine samples. Nisell et al. [58] found that blood sampling for assessment of renal function and 24 hour urine collections for measurement of albumin had little added value in anticipating the risk for developing maternal complications of pre-eclampsia. However, in light of the pathophysiologic progression of the disease process, the occurrence of oliguria is highly predictive of the risk of maternal mortality (RR 5.39; 95% CI 1.80 – 10.69; [59]). Data from the present study also indicate that oliguria occurs with some high degree of frequency (4.72% of aggregate women).

Similarly, there should be little concern about the impact on clinical laboratory resources for measurement of serum magnesium levels. Studies that correlated serum magnesium levels with clinical indicators conclude that these measurements should be limited to cases in which clinical indicators, identified through vigilant patient monitoring, suggest toxicity [27,60]. Thus, the concerns about the inability to provide the necessary nursing care to women undergoing magnesium sulfate therapy, and the increased use of laboratory resources appear to be unfounded.

## Conclusion

There is strong, evidence-based, global support for early identification and prompt treatment of women who develop pre-eclampsia, in order to promote maternal and newborn survival. Management approaches have been widely tested, and magnesium sulfate has emerged as the drug of choice. The concerns that are expressed about the safety of use of this drug, should be mitigated by findings from this integrative review that indicate a low incidence of the most severe side effects, documented in studies that used a wide variety of standard and modified drug regimens. The findings demonstrate that across all the included studies the adverse effects that are often of concern to providers occur infrequently, and that when adverse effects occurred, delay of repeat administration was generally sufficient to mitigate the effect.

While every maternal death is regrettable, maternal mortality directly attributable to the use of magnesium sulfate reported in these studies was extremely rare. Early screening and diagnosis of the disease, appropriate treatment with proven drugs, and reasonable vigilance for women under treatment should be adopted as global policy and practice.

## Competing interests

Each of the co-authors declares that we have no known conflict of interest regarding this manuscript.

## Authors’ contributions

JMS, RFL and SMC developed the ideas for the paper; RFL and EF-K conducted the literature search and review of the citations; EF-K and LH conducted the data extraction; JF and RL led the writing of the manuscript, with comments and contributions from JMS and SMC. All authors read and approved the final manuscript.

## Pre-publication history

The pre-publication history for this paper can be accessed here:

http://www.biomedcentral.com/1471-2393/13/34/prepub
